# Association between Crystalline Silica Dust Exposure and Silicosis Development in Artificial Stone Workers

**DOI:** 10.3390/ijerph18115625

**Published:** 2021-05-25

**Authors:** Mar Requena-Mullor, Raquel Alarcón-Rodríguez, Tesifón Parrón-Carreño, Jose Joaquín Martínez-López, David Lozano-Paniagua, Antonio F. Hernández

**Affiliations:** 1 Department of Nursing, Physiotherapy and Medicine, University of Almería, 04120 Almería, Spain; mrm047@ual.es (M.R.-M.); tpc468@ual.es (T.P.-C.); dlozano@ual.es (D.L.-P.); 2 Andalusian Council of Health and Families at Almería Province, 04005 Almería, Spain; josej.martinez@juntadeandalucia.es; 3 Department of Legal Medicine and Toxicology, University of Granada School of Medicine, 18016 Granada, Spain; ajerez@ugr.es; 4 Instituto de Investigación Biosanitaria, Granada (ibs.GRANADA), 18012 Madrid, Spain; 5 Centro de Investigación Biomédica en Red de Epidemiología y Salud Pública (CIBERESP), 18080 Madrid, Spain

**Keywords:** artificial stone, respirable crystalline silica, silicosis, occupational exposure, pulmonary disease, occupational epidemiology

## Abstract

Occupational exposure to respirable crystalline silica (SiO_2_) is one of the most common and serious risks because of the health consequences for the workers involved. Silicosis is a progressive, irreversible, and incurable fibrotic lung disease caused by the inhalation of respirable crystalline silica dust. A cross-sectional epidemiological study was carried out to assess the occupational risk factors that may contribute to the onset of silicosis in workers carrying out work activities with the inhalation of silica compact dust. The study population consisted of 311 artificial stone workers from the province of Almeria (southeast of Spain). Among them, 64 were previously diagnosed with silicosis and the rest of the participants (n = 247 workers) were not diagnosed with such a disease. The workers showing a greater risk of developing silicosis were those who installed kitchen worktops at consumers’ homes, as they did not use face-masks or were not provided with personal protective equipment (PPE) by their business. The results of this study provide support for the evidence indicating that silicosis is a major emerging health concern for workers in the artificial stone sector. Exposure to crystalline silica dust thus can influence the development of silicosis in those cases where individual and collective protection measures are not used or adequately applied.

## 1. Introduction

Occupational exposure to crystalline silica dust (SiO_2_) has become one of the most common and serious hazards for artificial stone workers. According to recent studies, a large number of workers are exposed to this mineral dust throughout the world, with more than 3.2 million workers in Europe and more than 2 million in the United States [1,2,3]. The main industries where occupational exposure to crystalline silica dust occurs include construction, mining, quarrying and stone crushing operations, foundries, brick making, concrete, ceramics and glass [4].

Silicosis is a progressive, irreversible, and incurable fibrotic lung disease that occurs due to the prolonged inhalation of respirable crystalline silica dust (RCS) [5]. The major risk factors for developing silicosis include cumulative lifetime exposure, total amount of inhaled RCS, and individual susceptibility [6,7]. When RCS is inhaled, particles can reach the lower respiratory tract and gas exchange areas (i.e., alveolar spaces) where they interact with lung tissue ultimately developing fibrotic nodules and scarring around the trapped silica particles. This fibrotic condition of the lung is called silicosis [5,8,9]. The manufacture and processing of artificial stone (AS) have been considered as possible sources of occupational exposure to high levels of RCS [10].

The content of silica in AS is approximately 90%, which is a much higher percentage than that of granite stones (30%) or natural marble (3%) [11]. The use of powerful devices for cutting and grinding artificial stone can cause high levels of exposure to RCS. However, not much information is available regarding the concentrations of silica dust generated in these tasks [12,13,14].

The inhalation of crystalline silica dust constitutes a risk factor for workers’ health. Exposed workers can suffer not only certain cardiovascular diseases, but also pulmonary tuberculosis, silicosis, chronic obstructive disease, some types of neoplasms, autoimmune diseases and kidney disorders [4,5,15]. The higher the exposure to silica dust, the greater the risk of mortality [4,16,17,18], thus making silica dust one of the greater public health problems.

The Occupational Safety and Health Administration (OSHA) and the National Institute for Occupational Safety and Health (NIOSH) identified silica exposure as a “health hazard for workers involved in manufacturing, finishing, and installation of natural and manufactured stone countertops products, both in fabrication shops and during in-home finishing and installation” [19].

Several studies reported outbreaks of silicosis among AS workers [20,21,22,23,24,25,26,27,28,29]. The growing concern for the scientific community is not only the higher incidence of the disease in workers exposed to AS, but also the different pathological features and the high degree of severity of silicosis associated with AS. Most epidemiological and clinical studies reported cases of accelerated silicosis characterized by a short latency period, extensive lung damage, and onset in young workers. The increased aggressiveness of silicosis associated with AS is usually attributed to the lack of adequate preventive or protective measures, which may be a plausible explanation given the high levels of exposure that occur in a short period of time.

Therefore, the aim of this study was to assess the possible occupational exposure factors leading to the development of silicosis in artificial stone workers exposed by inhalation to silica compact dust.

## 2. Material and Methods

### 2.1. Study Design and Geographical Location

A cross-sectional epidemiological study was conducted on artificial stone workers occupationally exposed to silica compact dust with the aim of analyzing the occupational exposure factors that may be involved in the occurrence of silicosis.

**Figure 1:**
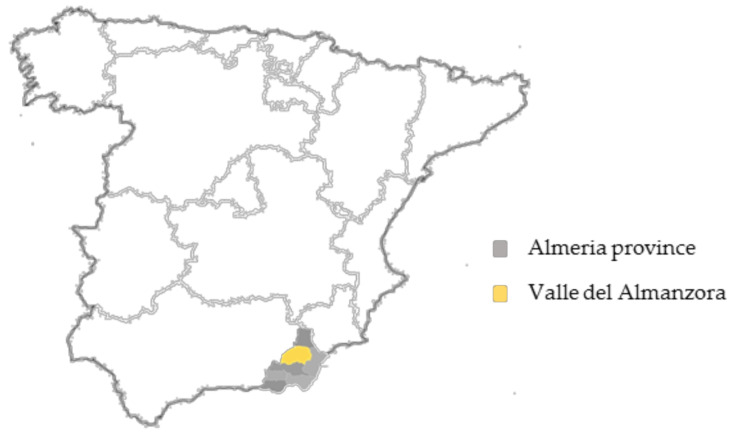
Geographic distribution of the study area (yellow) in the southeast of Spain (grey).

The study area was the Valle del Almanzora (Almanzora Valley), province of Almería (Southeastern Spain), where the majority of companies dedicated to the creation of artificial stone products are located (Figure 1). Almanzora Valley is characterized by having an over three-thousand-year-old marble industry that has nurtured world heritage by supplying white marble to numerous historical monuments.

### 2.2. Study Population and Data Collection

The study population consisted of 311 workers in the stone sector handling artificial stone or silica compacts that were exposed to crystalline silica dust on a daily basis. Participants were recruited during their scheduled annual occupational health surveillance program by the Occupational Risk Prevention Services (ORPS) of different companies located in the study area, and consisted of 64 workers with a previous diagnosis of silicosis and 247 workers lacking a diagnosis of silicosis. Study participants with a diagnosis of silicosis were declared as occupational diseases using the official notification system called CEPROSS (Comunicación de Enfermedades Profesionales en la Seguridad Social—Communication of Occupational Diseases to the Social Security) during the study period (2006–2019). CEPROSS is a program for the compulsory declaration of occupational diseases in Spain. Clinical information was provided by the Ministry of Employment, Training and Autonomous Work of the Junta de Andalucía for the study period. For each worker in the artificial stone sector with a diagnosis of silicosis, and notified through CEPROSS, three workers were recruited from the same sector but without silicosis, as a comparison group.

Those workers who volunteered to participate in the study signed an informed consent form after being informed about the objectives of the study and their right to drop from the study at any time. This study was approved by the Ethics and Research Commission of the Department of Nursing, Physiotherapy and Medicine of the University of Almeria (ID: EFM94/2021). All the procedures were performed in accordance with the ethical standards of the Helsinki Declaration.

Medical records were provided by the ORPSs of the companies involved in the study. The information collected was grouped into sociodemographic variables (age, educational level, smoking habit), respiratory symptoms (previous chronic diseases and the presence of respiratory symptoms), and occupational data (job information and description, workplace, accessibility and the proper use of personal protective equipment (PPE), etc.).

### 2.3. Data Analysis

Data were analyzed using the IBM SPSS statistical software package (SPSS 26.0 for Windows). A database was created with the collected information. A descriptive analysis of the continuous variables was carried out using means and standard deviations, whereas absolute and relative frequency distributions were calculated for categorical variables. The Kolmogorov–Smirnov test was used to assess the normality of continuous variables. Subsequently, continuous variables were compared by silicosis diagnosis using the Mann–Whitney *U* test as they were not normally distributed. Categorical variables were compared using the Chi-square test.

Multiple binary logistic regression analysis was used to assess the risk of having silicosis adjusted for variables that were considered to influence the statistical model based on the bivariate analysis. The level of statistical significance was established for a value of *p* < 0.05.

## 3. Results

A total of 311 male workers engaged in activities involving exposure to crystalline silica dust participated in the study. These workers were divided into two groups, separating those who had a previous diagnosis of silicosis (n = 64) and those lacking a silicosis diagnosis (n = 247).

The study groups did not differ in relation to possible confounding factors such as age, smoking, smoking time (years), and number of cigarettes smoked per day (Table 1). All participants were of Spanish nationality and most had basic studies (silicosis: 68.8%, n = 44; non-silicosis: 53.8%, n = 53.8%; *p* < 0.003).

The most frequent clinical form of the disease was acute silicosis (42.2%) followed by accelerated silicosis (32.8%) and chronic silicosis (25.0%) (Table 2). No significant differences were observed in relation to chronic diseases associated with silicosis among workers with or without silicosis. Chronic respiratory diseases, such as asthma and seasonal allergy, were the most frequent ones which were observed in 43.7% of the workers with silicosis and in 38.9% of those without silicosis.

Near-significant differences were observed according to the level of dyspnea. Among workers with silicosis, 25.1% presented small effort dyspnea and 32.4% great effort dyspnea. For workers without silicosis, 39.1% had dyspnea on little exertion and 26.6% had dyspnea on great exertion. Most of the workers did not have chronic obstructive pulmonary disease (COPD) (65.6 of workers with silicosis and 76.9% of workers without silicosis) (Table 2).

In relation to occupational data, all workers were exposed to crystalline silica dust 8 h a day, with the average number of years exposed being 8.40 (6.75) years for workers with silicosis, and 14.75 (9.47) years for workers without silicosis (Table 3). While 20.3% of the workers with silicosis carried out their work in the home of clients assembling kitchen countertops, 91.1% of the workers without silicosis carried out their activity in manufacturing workshops (cutting, processing, polishing and honing), with the differences being statistically significant (*p* < 0.01). No statistically significant differences were observed between the two groups in relation to job position performed, with cutting being the most common work activity performed (35.9% of workers with silicosis and 33.6% of workers without silicosis) (Table 3).

Most of the workers had received training in occupational risk prevention (ORP) (60.9% or workers with silicosis and 72.5% of workers without silicosis), although the differences were not statistically significant. Workers’ company provided most of them with PPE (64.1% in workers with silicosis and 80.2 in works without silicosis). Most of workers used face mask as PPE during the working day (84.4% of the workers with silicosis and 97.2% in those without silicosis), with the differences being statistically significant (*p* < 0.001) (Table 3).

Table 4 shows the results of the multiple binary logistic regression analysis of the possible factors that may be associated with the development of silicosis. Models were adjusted for the following independent variables: age, educational level, years worked with exposure to SiO_2_, workplace, PPE provided by the company and use of face mask as PPE. An increased risk of developing silicosis was observed for workers having no studies, for those working in the customers’ homes assembling kitchen countertops, those for whom PPE were not provided by their company and those who did not use face mask during the working day. For the variable time exposed to SiO_2_, an OR = 0.77 (*p* = 0.001) was obtained.

## 4. Discussion

The number of cases of silicosis associated with exposure to silica dust in artificial stone factories, as well as its subsequent assembly in homes, has raised great concern in recent years [25]. The present study analyzed the occupational factors that may contribute to the development of silicosis in artificial stone workers performing activities with the inhalation of silica compact dust.

### 4.1. Cases of Silicosis and Respiratory Symptoms

This study presents 64 workers diagnosed with silicosis from a total of 311 workers exposed to silica dust during a 14-year period (2006–2019). This figure is consistent with previous studies showing a high incidence of silicosis in artificial stone workers [10,24,25,26,27,28,29,30,31,32,33] because of the worldwide use of artificial stone products with high silica content, which can result in severe forms of silicosis.

According to The Spanish Institute of Silicosis, 219 new cases of silicosis were diagnosed in Spain in 2019. The profile of new diagnosed cases was featured by young active workers with shorter risky employment histories than those traditionally observed. In fact, the analysis of the evolution of silicosis cases in the last 15 years showed a remarkable increase in the number of cases in the latter 2 years, reaching similar incidence as to that of 2010 and 2011, when a notable increase in cases was observed as a consequence of the economic crisis [33].

The mean age of workers diagnosed with silicosis of the present study (39 years) is consistent with other studies performed in other Andalusian provinces, which showed the mean age of the patients to be 39.81 years [30]. However, other studies also carried out on Andalusian workers reported a lower mean age: 33 years for a study carried out in Cádiz [29], and 34 years for another one conducted in Seville [32].

In regard to respiratory symptoms, this study found that 25.1% of AS workers showed small effort dyspnea and 32.4% great effort dyspnea, while almost half of the workers with silicosis had respiratory symptoms. These findings are in agreement with those from other studies, such as Perez et al. [29], which reported mild respiratory symptoms in 82.6% of the 46 workers diagnosed with silicosis, whereas the rest were asymptomatic; however, all felt more tired than usual before diagnosis. In addition, Hoy et al. [10] reported seven workers involved in dry cutting artificial stone and exposed to silica dust that had a cough and shortness of breath on exertion.

This study found no differences between the workers with or without a diagnosis of silicosis regarding respiratory alterations such as dyspnea, COPD and other chronic respiratory pathologies such as asthma and seasonal allergy. When respirable silica particles are inhaled, they can reach the lower respiratory tract and gas exchange zones where, after being phagocytosed by alveolar macrophages, they can persist and trigger an inflammatory process characterized by the production of reactive oxygen species. The inflammation generated by these highly reactive molecules damages the lung parenchyma eventually leading to respiratory disorders (e.g., dyspnea and COPD). All workers exposed to silica dust, regardless of whether or not they develop silicosis, may present respiratory disorders as a result of lesions in the lung parenchyma [34].

### 4.2. Occupational Exposure Factors to Silica Dust

#### 4.2.1. Exposure Time and Job Position

As mentioned above, the several studies have recently reported cases of silicosis related to exposure to silica dust. Short latency periods, between 4 and 10 years, were described before the development of the disease, which may be related to a higher level of exposure to silica dust as a result of tasks carried out in manufacturing workshops (cutting, polishing) and during the installation of countertops at customers’ homes [10,24]. These data are consistent with the results of this study, in which workers had been exposed to silica dust for an average of 8 years. Among the workers with silicosis tasked with assembling kitchen countertops at customers’ homes, 20.3% presented almost twice the risk of developing silicosis as compared to those who performed their activity in manufacturing workshops. Among them, the most affected workers were assemblers, who carried out their activity with stone cutting machines without wet cutting function.

In Australia, Calver et al. [4] reported an exposure time to silica dust between 4 and 10 years and workers also performed dry cutting inside the workshops. Shtraichman et al. [24] reported that artificial stone workers were exposed for at least six years to silica dust. In another study also conducted in Israel, Kramer et al. [26] reported a longer exposure time, between 17 and 22 years, mainly affecting workers performing the dry cutting of artificial stone.

In Spain, several studies reported different times of exposure to silica dust for their workers [29,30,31,32]. Perez et al. [29,32] reported an average of 11 years for their workers involved in the manufacture and subsequent assembly of kitchen countertops. Pascual et al. [30], described a very similar time of exposure for their workers, around 12 years. The main tasks they performed was cutting and polishing in the workshop and also the assembly of countertops at customers’ homes. Another Spanish study reported 11 years of mean exposure for workers handling artificial quartz aggregates as assemblers/cutters/sanders of countertops, whereas the rest of individuals working in companies with no artificial quartz aggregates exposure had an average exposure time of 27 years [31].

Strikingly, the workers assembling artificial stone pieces of kitchen countertops at customers’ homes had a higher risk of having silicosis than workers carrying out their job at the factory. This observation has previously been raised by Perez et al. [29,32] who found a higher prevalence of silicosis in workers installing kitchen countertops, likely because the use of PPE was lacking when carrying out assembly tasks at customers’ homes. In addition, wet cut to minimize dust generation, which represents the main prevention technique in kitchen countertop manufacturing companies, is not feasible at customers’ homes.

#### 4.2.2. Smoking Habit

Several years ago, smoking was considered as a risk factor for the development of silicosis in workers exposed to silica dust [35]. Even low exposure levels of respirable crystalline silica have been associated with lung cancer [36]. Moreover, several studies have shown that smoking can increase the harmful effect of exposure to silica dust [37,38]. Likewise, different studies have reported that the interaction of silica exposure and smoking was associated with an increased risk of mortality [39,40]. However, the present study found no significant differences between diagnosed cases of silicosis and smoking habit, which is in agreement with Pascual et al. [30].

### 4.3. Use of Personal Protective Equipment (PPE)

Regarding the use of PPE, 64% of workers with silicosis were provided PPE by their company. Nevertheless, they had a three-fold higher risk of developing silicosis as compared to workers who were not provided PPE by their companies, and four times more risk in workers who did not wear mask. However, another similar study carried out in Spain. Reference [29] reported that most of the companies did not provide PPE to their workers and when they did so, workers’ PPE was not used correctly.

Furthermore, 84% of workers diagnosed with silicosis reported the use of PPE (masks). However, for most of the studies reviewed, workers reported they failed to wear PPE. Thus, in the study of Kramer et al. [26], workers did not use any type of PPE for an average of 10 to 12 h per day. In Spain, Perez et al., reported that only 32.6% of workers wore PPE (mask, glasses, gloves, special footwear and a helmet) [29]. In another study, workers who performed their work as home fitters reported they were not provided any PPE [30]. In an Australian study, only a very small percentage of workers had access to protective equipment [10]. Therefore, workers who were not provided PPE or who did not use it correctly are at an increased risk of developing silicosis, as occurs with countertop assemblers.

### 4.4. Information and Training of Workers

The results of this study indicate that 61% of workers with silicosis reported to have received some occupational risk-prevention (ORP) training course at any time. The regression analysis showed that workers with no education level had an eight-fold greater risk of developing silicosis. It is important to note that adequate information and training for workers regarding exposure to silica dust during their work activities, as well as better knowledge about the risks derived from exposure to silica dust, may be partly to account for the adequate protection of workers included in this study.

### 4.5. Strengths and Limitations

The main strength of this study was the conduct of a multivariate regression analysis adjusted for occupational variables and their possible influence on the development of silicosis in which two groups of workers exposed to silica dust were compared, one with silicosis and another without silicosis. To date, only a few studies performed a similar analysis. Another strength of this study was the relatively higher number of participants included with respect to other similar studies published to date. However, the main limitation is that it is a cross-sectional study, which does not allow causal relationships to be drawn and the lack of follow-up of workers over time.

## 5. Conclusions

The results of this study indicate that exposure to crystalline silica dust in workers in the artificial stone sector contributes to the development of silicosis in those cases where individual and collective protection measures are not used or not applied correctly. The main risk group is made up of assemblers who installed artificial stone pieces of kitchen countertops at consumers’ homes, as they are exposed to high levels of crystalline silica dust during this work task.

Hence, companies and occupational risk prevention services should be aware of this problem and play an active role in the evaluation of preventive measures, as well as in the identification and control of exposure to silica dust, especially in activities and workplaces with a greater risk, such as the assembly of kitchen countertops outside of their companies, at the customers’ homes.

## Figures and Tables

**Table 1:** Comparison of sociodemographic data (age and education level) and smoking habit between the two study groups, “silicosis” and “no silicosis”.

Characteristic		Silicosis (n = 64)	No Silicosis (n = 247)	*p* Value
Age, in years		39.03 (7.90)	40.13 (11.88)	0.37 *
Educational level	Non studies	12 (18.8%)	26 (10.5%)	0.003 **
	Low	44 (68.8%)	133 (53.8%)	
	Medium	6 (9.4%)	77 (31.2%)	
	High	2 (3.1%)	11 (4.5%)	
Tobacco	Yes	33 (18.1%)	149 (81.9%)	0.07 **
	No	10 (16.7%)	50 (83.3%)	
	Former smoker	21 (69.6%)	48 (30.4%)	
No. Cigarettes/day		22.35 (9.72)	21.21 (11.02)	0.57 **
Smoking time (in years)		22.30 (10.85)	21.13 (11.43)	0.59 **

*p* value obtained using * Mann–Whitney *U* test for continuous variables or ** Chi-squared test for categorical variables.

**Table 2:** Comparison of the respiratory symptoms data between the two groups, “silicosis” and “no silicosis”.

Characteristic		Silicosis (n = 64)	No Silicosis (n = 247)	*p* Value
Chronic Pathologies	None	14 (21.9%)	92 (37.2%)	0.15 *
	Cardiovascular	10 (15.6%)	29 (11.7%)	
	Endocrine–metabolic	9 (14.1%)	19 (7.7%)	
	Respiratory ^1^	28 (43.7%)	96 (38.9%)	
	Urological	3 (4.7%)	11 (4.5%)	
Dyspnea	Dyspnea (little exertion)	25 (39.1%)	62 (25.1%)	0.08 *
	Dyspnea (great exertion)	17 (26.6%)	80 (32.4%)	
	No	22 (34.4%)	105 (42.5%)	
COPD	Yes	57 (34.4%)	57 (23.1%)	0.06 *
	No	42 (65.6%)	190 (76.9%)	
Silicosis Clinic	Acute	27 (42.2%)	-	-
	Accelerated	21 (32.8%)	-	
	Chronic	16 (25.0%)	-	

*p* value obtained with * Chi-squared test; ^1^ seasonal allergy and asthma.

**Table 3:** Comparison of occupational data between the two groups of workers.

Characteristic		Silicosis (n = 64)	No Silicosis (n = 247)	*p*-Value
Time Worked with Exposure to SiO_2_ (years)		8.40 (6.75)	14.75 (9.47)	0.001 *
Job	Court	23 (35.9%)	83 (33.6%)	0.07 **
	Elaboration	13 (20.3%)	64 (25.9%)	
	Polished	8 (12.5%)	51 (20.6%)	
	Honed	7 (10.9%)	27 (10.9%)	
	Setter	13 (20.3%)	22 (8.9%)	
Workplace	Assembly at customers’ homes	13 (20.3%)	22 (8.9%)	0.01 **
	Manufacturing workshops	51 (79.7%)	225 (91.1%)	
Trained in ORP	Yes	39 (60.9%)	179 (72.5%)	0.07 **
	No	25 (39.1%)	68 (27.5%)	
PPE provided by the company	Yes	41 (64.1%)	198 (80.2%)	0.007 **
	No	23 (35.9%)	49 (19.8%)	
Use the mask	Yes	54 (84.4%)	240 (97.2%)	0.001 **
	No	10 (15.6%)	7 (2.8%)	

*p* value obtained with * Mann–Whitney *U* test or ** Chi-squared test.

**Table 4:** Stepwise multiple binary logistic regression analysis of the risk of developing silicosis adjusted for a number of potential risk factors.

Parameters	OR	95% C.I.	*p*-Value
Age (in years)	1.13	1.06–1.19	0.001
Educational levels (no studies)	8.51	1.25–13.78	0.001
Time exposed to SiO_2_ (years)	0.77	0.71–0.83	0.001
Workplace (assembly at customers’ homes)	2.69	1.13–6.41	0.02
PPE provided by the company (no)	3.64	1.49–8.91	0.05
Use the face mask (no)	4.06	1.09–15.03	0.03

The regression model was adjusted for age, educational levels (0: high; 1: medium, 2: low, 3: no-studies), time exposed to SiO_2_ (years), workplace (0: manufacturing workshops; 1: assembly at home), PPE provided by the company (0: yes; 1: no), use the mask (0: yes, 1: no).
